# Risk Factors Associated With Tuberculosis Diagnostic Delay in the Jiangsu Province, China (2011-2021): Spatiotemporal Database Analysis Study

**DOI:** 10.2196/80052

**Published:** 2026-01-26

**Authors:** Yifan Tang, Cheng Chen, Mingming Chen, Kai Wang, Sifan Wang, Yi Lin, Qiao Liu, Chengxiu Ling, Tenglong Li, Limei Zhu

**Affiliations:** 1 Department of Biostatistics, Academy of Pharmacy, Xi’an Jiaotong-Liverpool University, Suzhou, China; 2 Department of Mathematical Sciences, University of Liverpool, Liverpool, United Kingdom; 3 Department of Chronic Communicable Disease, Center for Disease Control and Prevention of Jiangsu Province, No.172 Jiangsu Road, Gulou District, Nanjing, China, (025)83759455-8

**Keywords:** tuberculosis, diagnostic delay, Bayesian spatiotemporal model, Moran *I* index, integrated nested Laplace approximation

## Abstract

**Background:**

Tuberculosis (TB) remains a major public health concern. Despite improved diagnostic tools, delays in TB diagnosis persist and hinder control efforts.

**Objective:**

This study aims to investigate the spatiotemporal patterns of TB diagnostic delay and identify individual and spatial risk factors in Jiangsu Province, China, from 2011 to 2021.

**Methods:**

This study included 332,091 patients with TB who reported in Jiangsu Province from 2011 to 2021, using data obtained from the Jiangsu TB Information Management System, and diagnostic delay was defined as an interval of more than 28 days between symptom onset and diagnosis. Logistic regression was used to evaluate individual-level factors associated with delayed status, while a Bayesian spatiotemporal Beta model was used to analyze county-level TB diagnostic delay rates and assess spatial correlation using the global Moran *I*. The panel Granger causality analysis explored the temporal dynamics of delay rate transitions.

**Results:**

Male patients, educators, and those diagnosed at the local Centers for Disease Control and Prevention had lower odds of diagnostic delay, whereas the older adults, agricultural workers, migrants, clinically diagnosed cases, and those diagnosed at community health centers had higher odds of delay. Spatial clustering in TB diagnostic delay rates was significant from 2015 onward (Moran *I*=0.110-0.193; all *P*<.05), excluding 2018 when Moran *I* was 0.054. The Bayesian spatiotemporal Beta model, which accounted for 31.8% of the total variation due to spatial structure, indicated that for each 1-unit increase in the proportion of local patients and for each 100,000-person increase in resident population, the TB diagnostic delay rate decreased by 33.9% (95% CI 0.128-0.498) and 2% (95% CI 0.005-0.033), respectively. The panel Granger causality analysis indicated that TB incidence and health care technicians significantly influenced temporal changes in delay rates.

**Conclusions:**

TB diagnostic delays in Jiangsu were influenced by both individual and spatial factors, with the proportion of local patients and resident population size contributing significantly to spatiotemporal variation. Tailored interventions targeting high-risk groups and health care settings are needed.

## Introduction

Tuberculosis (TB) is a chronic infectious disease caused by *Mycobacterium tuberculosis* and is primarily transmitted via airborne particles [1]. In 2022, TB was the second leading cause of death from a single infectious agent, surpassed only by COVID-19 [2]. Globally, an estimated 10.8 million new TB cases occurred in 2023, with an incidence rate of 134 per 100,000 population, posing a grave threat to public health [3]. As the country with the third highest TB burden [4], China has made significant efforts in TB control over recent decades, increasing the case detection rate from 30% in the 1990s to 80% by 2005 and thereby effectively reducing transmission and incidence [5,6]. In Jiangsu Province, China, TB remains a critical public health challenge, consistently ranking second among reported class A and B infectious diseases [7].

The World Health Organization (WHO) End TB Strategy emphasized the importance of early diagnosis and timely treatment for TB control and prevention, as well as reducing treatment costs [8]. However, most national TB control programs primarily rely on passive case finding, a practice often resulting in treatment delays exceeding 1 month in approximately 42% of patients [9]. The extent of these delays varies globally due to socioeconomic and health care disparities, with particularly severe delays observed in less developed regions. For instance, reported median total delays range significantly from 68 days in France to 104 days in Ghana and up to 366 days in Afghanistan [10]. Therefore, identifying the key factors contributing to delays in specific regions is essential for developing targeted TB interventions and control measures.

Extensive research has examined risk factors contributing to TB diagnostic delays, which arise from complex interactions of individual behaviors, social determinants, and health care system challenges [11]. The declining clinical awareness of TB among health care workers, especially in low-incidence settings, combined with the nonspecific nature of typical TB symptoms, such as persistent cough and sputum production, often leads to early misdiagnosis as common respiratory infections [12]. Meanwhile, misdiagnosis and missed cases are exacerbated by urban-rural disparities in medical resources and surges in diagnostic pressure during peak health care demand periods, such as holidays or influenza seasons [4]. Socioeconomically vulnerable populations, including migrant workers and older adults, often experience delays in seeking medical care due to limited access to health care insurance, language barriers, and varying levels of education [13,14].

Although previous analyses have identified key risk factors, they have generally failed to sufficiently account for the spatial and temporal dependence inherent in TB diagnostic delays [15,16]. Past research using descriptive statistics has highlighted spatial heterogeneity, for instance, by revealing median delays of 30 days in eastern or central China versus 41 days in the west [6] and identifying regional disparities in Portugal [17]; these studies often overlook spatial autocorrelation. To address this limitation, the Bayesian spatiotemporal model provides a rigorous framework that incorporates explanatory variables to capture large-scale trends while also accounting for residual dependencies to reveal robust spatial patterns and potential risk factors [18]. Furthermore, by integrating prior knowledge to quantify uncertainty, this approach enhances both the accuracy and interpretability of findings [19,20]. The integrated nested Laplace approximation (INLA) algorithm offers an efficient approach for implementing Bayesian inference in such complex models [21]. Notably, the spatiotemporal patterns of TB diagnostic delay have been rarely investigated using this Bayesian approach, representing a critical research gap that this study aims to address.

This study used Bayesian spatiotemporal analysis to investigate TB diagnosis delays, capturing spatiotemporal dependencies and examining patterns of temporal and spatial variation. We have the following three research goals: (1) assess the existence of spatial and temporal autocorrelation in TB diagnostic delays within Jiangsu Province, China; (2) identify individual-level risk factors associated with delayed diagnosis; and (3) determine county-level determinants of diagnostic delay rates, while accounting for potential temporal and spatial random effects.

## Methods

### Data and Variables

We obtained TB surveillance data from the Jiangsu Tuberculosis Information Management System (TBIMS), spanning January 1, 2011, to December 31, 2021. The original dataset contained 354,274 infection cases reported in Jiangsu Province during this period, including patient information such as names, ages, sex, occupations, sources of patients, types of diagnosis, tracking status, types of hospital, dates of birth, onset dates, diagnostic dates, and so on. A total of 332,091 patients were analyzed in this study following exclusion criteria: (1) patients diagnosed and reported between January 1, 2011, and December 31, 2021, at health care institutions outside Jiangsu Province (n=8010); (2) patients with missing critical information (n=12,889); and (3) patients whose standardized *z* value for the total diagnostic delay that exceeded 3 (ie, more than 3 SD from the mean; n=1266) [22].

Total diagnostic delay, defined as the interval from the onset of TB symptoms to formal diagnosis, comprises both patient delay and health system delay [13,23]. Patient delay refers to the time between symptom onset and the first medical consultation, while health system delay spans from the time of the first health care visit to diagnosis. This study focused exclusively on total diagnostic delay, as the dataset lacked information regarding the date of the first medical visit. At the individual level, the outcome was defined as the diagnostic delay status, which indicated whether a patient’s total diagnostic delay exceeded 28 days, a commonly adopted threshold for total TB diagnostic delay based on previous studies [13]. At the county level, the outcome was the TB diagnostic delay rate, which was calculated by dividing the number of delayed patients (ie, those with more than 28 d of total diagnostic delay) by the total number of patients in each county each year.

In addition to the individual-level TB surveillance data from Jiangsu TBIMS, a total of 9 annual county-level explanatory variables were included, categorized into three distinct domains: (1) demographic factors, comprising the annual proportions of older adult patients (≥60 y), male patients, local patients, and agricultural-worker patients among reported cases; (2) socioeconomic and health care indicators, including gross domestic product (GDP) per capita (adjusted to the 2021 Consumer Price Index), resident population size, TB incidence rate (per 1000 population), and the health care technicians (professionals per 1000 population); and (3) pandemic period, a binary variable (1=2020‐2021; 0=2011‐2019) introduced to adjust for the potential impact of social isolation policies and health care resource diversion during the COVID-19 pandemic. Data regarding health care technicians, GDP, and resident population were sourced from annual county statistical yearbooks, while other variables were aggregated directly from the TB surveillance data.

### The Bayesian Spatiotemporal Model

To explore the spatial correlation of the TB diagnostic delay rate across 89 districts and counties in Jiangsu Province from 2011 to 2021, we calculated the global Moran *I* to measure the spatial correlation [24,25]. When the result for Moran *I* is statistically significant, a positive value for Moran *I* suggests spatial clustering, while a negative value suggests spatial dispersion. The closer the value of *I* is to 1 or −1, the stronger the spatial association, while a value near 0 indicates a random spatial distribution of TB diagnostic delay rates.

Prior to the spatiotemporal modeling, multivariable binary logistic regression was used to identify risk factors associated with individual diagnostic delay status (binary outcome: 1 if total delay >28 d, 0 otherwise). Subsequently, a logit-link Bayesian Beta regression model was then applied, incorporating fixed effects for the proportion of older adult patients, proportion of male patients, proportion of local patients, proportion of agricultural-worker patients, GDP, TB incidence rate, number of health care technicians, and resident population, along with spatial and temporal random effects.

Specifically, let y(s,t) denote the TB diagnostic delay rate in year *t* over district or county *s*, with values ranging from 0 to 1. Here, *t*=1, 2, ..., 11 represents the years 2011 to 2021, and *s*=1, 2, ..., 95 represents the 95 counties or districts in Jiangsu Province, China. We further assumed that y(s,t) follows a β distribution with mean μ(s,t)∈(0,1) varying over time across counties or districts, and a constant precision parameter ϕ§gt;0. Namely,


$$\mathrm{logit}\mu (s,t)=x^{T}(s,t)\beta +\delta_{t}+u_{s}+\epsilon (s,t),$$

where the vector x(s,t) represents the regional-level variables in Table 3. The ϵ(s,t) term is an unstructured random effect in the model. We employed the first-order Gaussian random walks (RW1) model and the Besag-York-Mollié 2 model to capture the overall temporal random effect $\delta_{t}$ and spatial random effect $u_{s}$ [26].

The Besag-York-Mollié 2 model was used to capture spatial random effects by combining structured and unstructured spatial components through a mixing parameter [27]. The spatial effect for area i is expressed as:


$$b_{i}=\frac{1}{\sqrt{\tau_{b}}}\left(\sqrt{1-\phi }v_{i}^{*}+\sqrt{\phi }u_{i}^{*}\right),$$

where $\tau_{b}$ is the overall precision, $u_{i}^{*}$ is a standardized intrinsic conditional auto-regressive component capturing structured spatial dependence, and $v_{i}^{*}$ is a standardized Gaussian noise term representing unstructured spatial variability. The mixing parameter ϕ∈[0,1], is a spatial smoothing parameter, measuring the proportion of the marginal variance explained by the structured random effect.

As a latent effect implemented in the R-INLA package, the first-order Gaussian random walk was used to model temporal dependence [26]. For a latent Gaussian field $u=(u_{1},\ldots ,u_{n}{)}^{T}$, it is a random walk of order 1 if the increments $\Delta u_{i}=u_{i}-u_{i-1}$ are independent and identically distributed Gaussian random variables with zero mean and precision τ§gt;0 (inverse variance).

Based on the delay rates estimated from the Bayesian spatiotemporal Beta model, we dichotomized at the median and then fitted a Bayesian spatiotemporal binomial model to identify factors associated with a higher likelihood of diagnostic delay. Furthermore, to explore the temporal dynamics and potential drivers of delay rate, we conducted a panel Granger causality analysis [28].

All statistical analyses were conducted using R software (version 4.4.1; R Foundation for Statistical Computing). The *INLA*, *stats*, and *lmtest* packages were used to conduct the Bayesian spatiotemporal modeling, logistic regression, and the Granger causality analysis, respectively, with default prior distributions specified for the INLA hyperparameters.

### Ethical Considerations

Anonymized data were obtained from the Jiangsu TBIMS, with all personal identifiers (eg, name and ID number) removed prior to analysis. The study protocol was reviewed by the ethical review board of the Jiangsu Provincial Center for Disease Control and Prevention (Jiangsu CDC) and granted an official exemption (acceptance: SL2025-B030-01), as the study was deemed retrospective and the data were deidentified. Data access and usage were strictly governed by a formal Data Usage Agreement between the Jiangsu CDC and the study authors. Informed consent was not required for this retrospective study using anonymized data.

## Results

### Descriptive Statistics

Table 1 summarizes the demographic and clinical features of patients with TB with delayed (≥28 d) and nondelayed (<28 d) diagnoses, respectively, in Jiangsu Province (2011-2021). Among all patients, the majority were male participants (239,692/332,091, 72.18%), working in agriculture (204,983/332,091, 61.72%), and reported by general hospitals (148,960/332,091, 44.86%), with a high proportion of local residents (239,440/332,091, 72.10%).

**Table 1.** Demographic and clinical characteristics of patients with tuberculosis reported in Jiangsu Province, 2011-2021.

Variables	Total delay days (<28), n (%)	Total delay days (≥28), n (%)
Age (y)		
<60	132,502 (39.90)	47,358 (14.26)
≥60	79,287 (23.87)	72,944 (21.97)
Sex		
Female	57,212 (17.23)	35,187 (10.60)
Male	154,577 (46.55)	85,115 (25.63)
Occupation		
Agriculture	128,957 (38.83)	76,026 (22.89)
Education	9219 (2.78)	4523 (1.36)
Health care	913 (0.27)	554 (0.17)
Housekeeping	36,705 (11.05)	20,111 (6.06)
Officials	2264 (0.68)	1415 (0.43)
Service	3660 (1.10)	1778 (0.54)
Worker	20,304 (6.11)	9386 (2.83)
Other	9767 (2.94)	6509 (1.96)
Source of patient		
Local	158,987 (47.87)	80,453 (24.23)
Different county	45,710 (13.76)	35,262 (10.62)
Different city	4471 (1.35)	3482 (1.05)
Different province	2621 (0.79)	1105 (0.33)
Types of diagnosis		
Confirmed	78,554 (23.65)	45,019 (13.56)
Clinically diagnosed	129,551 (39.01)	74,750 (22.51)
Suspected	3684 (1.11)	533 (0.16)
Tracking status		
Recorded	74,344 (22.39)	44,062 (13.27)
Referred	92,716 (27.92)	45,878 (13.81)
Tracked	32,184 (9.69)	23,132 (6.97)
Other	12,545 (3.78)	7230 (2.18)
Types of hospital		
Designated hospital	62,425 (18.80)	37,307 (11.23)
CHC^a^	28,793 (8.67)	15,460 (4.66)
CDC^b^	26,392 (7.95)	11,298 (3.40)
General hospital	93,216 (28.07)	55,744 (16.79)
Other	963 (0.29)	493 (0.15)
COVID-19		
Pre-epidemic	182,934 (55.09)	28,855 (8.69)
Epidemic	103,886 (31.28)	16,416 (4.94)

a CHC: community health center.

b CDC: Centers for Disease Control and Prevention.

**Figure 1.**
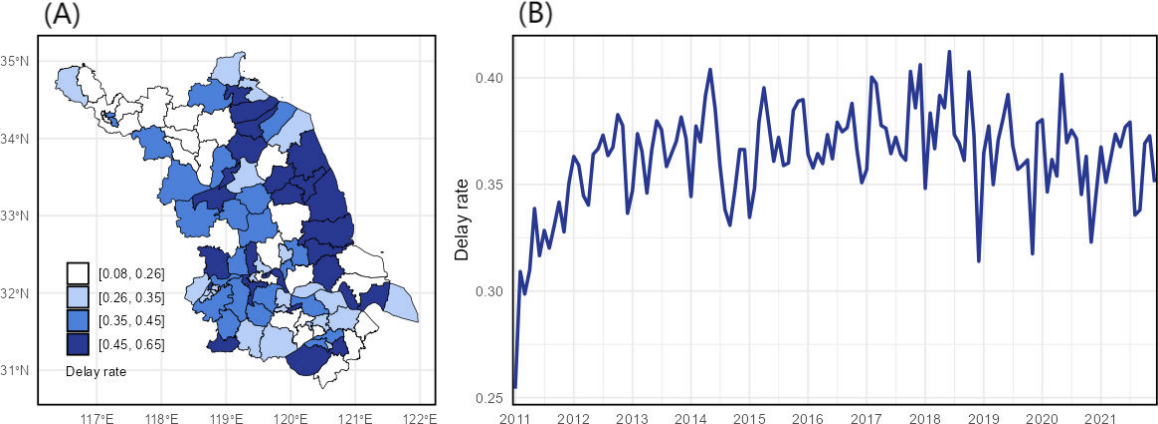
Annual average spatial distribution across 95 counties (A) and monthly provincial average temporal trends (B) of tuberculosis delayed diagnoses in Jiangsu Province, 2011-2021.

Figure 1A shows the spatial distribution of average TB diagnostic delay rates in Jiangsu Province over the period from 2011 to 2021. The cities of Yancheng and Huaian generally exhibited the highest delay rates, and the Binhai district in Yancheng recorded a peak delay rate of 93.35% in 2016. Figure 1B illustrates the temporal trend across all 95 counties. An increasing trend was evident from 2011 to 2014, followed by a plateau. Notable distinct declines in the average delay rate were observed in specific months, including December 2018, November 2019, and November 2020.

### Analysis at the Individual Level

**Figure 2.**
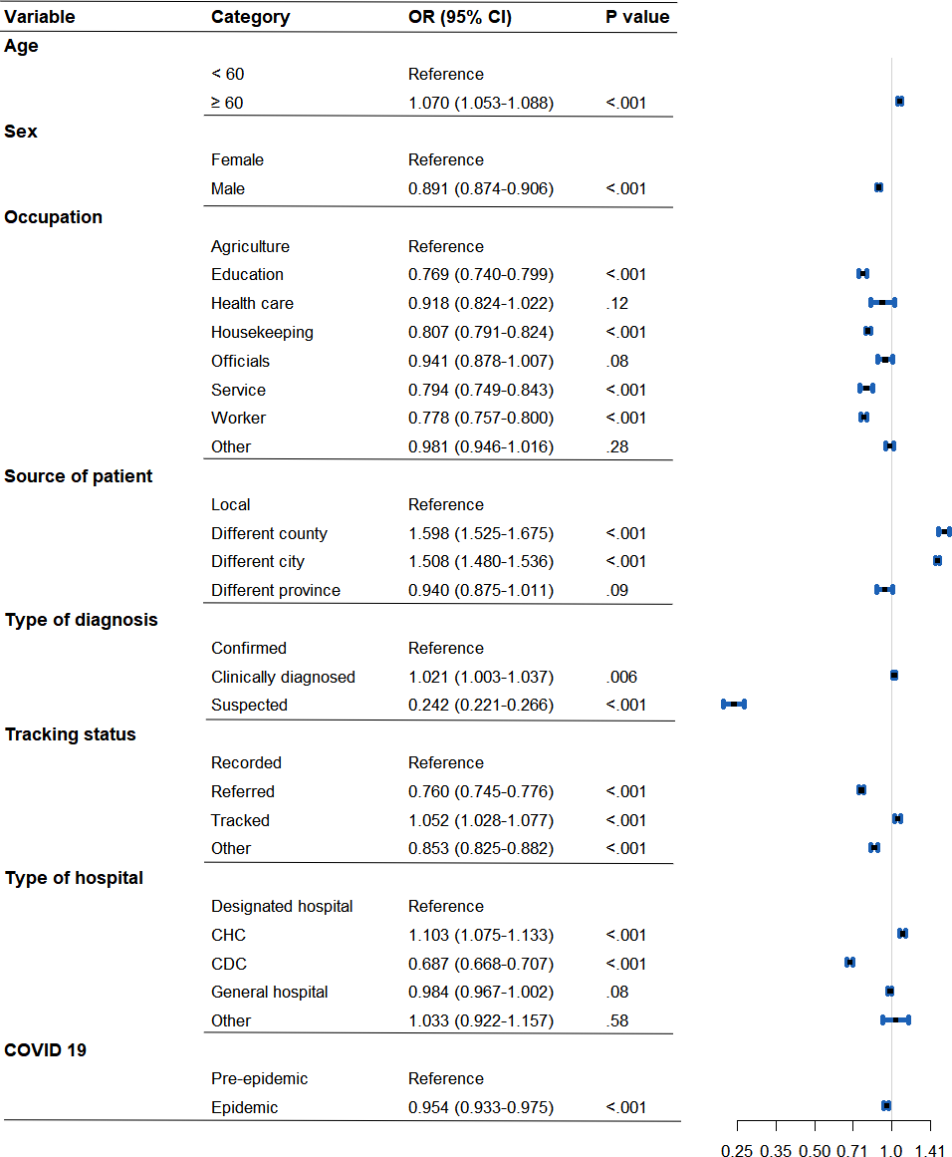
Odds ratios (ORs) from logistic regression model for factors affecting tuberculosis diagnostic delay status at the individual level in Jiangsu Province, 2011-2021. CDC: Centers for Disease Control and Prevention; CHC: community health center.

Figure 2 presents the results of a logistic regression analysis used to investigate potential risk factors associated with individual TB diagnosis delay status. Specifically, males had significantly lower odds of experiencing delayed TB diagnosis compared to females (odds ratio [OR] 0.891, 95% CI 0.874-0.906), while patients older than 60 years had slightly higher odds of delayed TB diagnosis compared to those who were younger than 60 years (OR 1.070, 95% CI 1.053-1.088). We also found that patients who worked in agriculture had higher odds of delayed TB diagnosis than patients in other occupations. Particularly, the differences between patients working in agriculture and patients of occupations in education (OR 0.769, 95% CI 0.740-0.799), housekeeping (OR 0.807, 95% CI 0.791-0.824), service (OR 0.794, 95% CI 0.749-0.843), and worker (OR 0.778, 95% CI 0.757-0.800) were statistically significant. Local patients (from the same county/district) had significantly lower odds of delayed TB diagnosis than patients from different counties (OR 1.598, 95% CI 1.525-1.675) or cities (OR 1.508, 95% CI 1.480-1.536) in Jiangsu. Regarding types of diagnosis, the suspected cases had significantly lower odds of delayed TB diagnosis compared to the confirmed cases (OR 0.242, 95% CI 0.221-0.266), while the clinically diagnosed cases had significantly higher odds of delayed TB diagnosis (OR 1.021, 95% CI 1.003-1.037) compared to the confirmed cases. Patients diagnosed at community health centers (CHCs) had higher odds of delayed diagnosis compared to those diagnosed at designated TB hospitals (OR 1.103, 95% CI 1.075-1.133). However, patients diagnosed at local CDCs had considerably lower odds of delayed diagnosis than those diagnosed at designated TB hospitals (OR 0.687, 95% CI 0.668-0.707). Finally, the COVID-19 period was associated with significantly lower odds of diagnostic delay (OR 0.954, 95% CI 0.933-0.975).

### Analysis at the County Level

Table 2 presents the global Moran *I* indices for TB diagnostic delay rates from 2011 to 2021. From 2011 to 2014, the Moran *I* values were close to zero (all *P*>.05), indicating no significant spatial autocorrelation. However, a marked increase in Moran *I* was observed from 2015 onwards, suggesting the emergence of substantial spatial clustering. Consequently, the Bayesian spatiotemporal Beta model was used to fully account for the spatial autocorrelation found in TB diagnostic delay rates across the years.

**Table 2.** Global Moran *I* index for tuberculosis diagnostic delay rate in Jiangsu Province, 2011-2021.

Year	Moran *I*	*P* value
2011	0.018	.33
2012	0.079	.08
2013	0.047	.19
2014	0.040	.22
2015	0.193	<.001
2016	0.167	.003
2017	0.161	.004
2018	0.054	.16
2019	0.113	.03
2020	0.110	.03
2021	0.085	.07

Table 3 presents the fixed effect estimates for the proportion of older adult patients, proportion of male patients, proportion of local patients, proportion of agricultural-worker patients, GDP, TB incidence rate, number of health care technicians, and resident population from the Bayesian spatiotemporal Beta model. For each 1-unit increase in the proportion of local patients, the TB diagnostic delay rate decreases by 33.9% (1−exp[−0.415], 95% CI 0.128-0.498). Additionally, each 100,000-person increase in resident population is associated with a 2% (95% CI 0.005-0.033) decrease in TB diagnostic delay. For random effects, the precision parameter $\tau_{b}$ was estimated as 3.411, indicating a moderate degree of spatial variation in the diagnostic delay rates across counties. In addition, the mixing parameter ϕ was estimated as 0.318, meaning that the structured spatial component accounts for 31.8% of the total spatial variation in TB diagnostic delay rate.

**Table 3.** The result of the Bayesian Beta spatiotemporal model of TB^a^ diagnostic delay rate at the county level in Jiangsu Province, 2011-2021.

Variables	Mean (SD)	0.025 quantile	0.975 quantile
Fixed effect			
Proportion of older adult patients (%)	0.234 (0.261)	−0.281	0.747
Proportion of male patients (%)	−0.002 (0.014)	−0.030	0.026
Proportion of local patients (%)	−0.415 (0.140)	−0.691	−0.138
Proportion of agricultural-worker patients (%)	0.187 (0.184)	−0.176	0.548
GDP^b^ (thousand yuan per person)	0.061 (0.037)	−0.014	0.135
TB incidence rate (%)	0.055 (0.062)	−0.068	0.180
Health care technicians (per 1000 residents)	−0.008 (0.008)	−0.024	0.007
Resident population (in 100,000)	−0.020 (0.007)	−0.034	−0.005
COVID-19 (0‐1)	−0.049 (0.050)	−0.148	0.053
Random effect			
Precision parameter for BYM2^c^	3.411	2.389	4.828
Mixing parameter for BYM2 (ϕ)	0.318	0.085	0.652

a TB: tuberculosis.

b GDP: gross domestic product.

c BYM2: Besag-York-Mollié 2.

Figure S1 in Multimedia Appendix 1 illustrates the spatiotemporal distribution of the estimated TB diagnostic delay rate obtained from the Bayesian spatiotemporal Beta model. Higher estimated delay rates were concentrated in the northern coastal cities such as Huaian, Yancheng, and Lianyungang, while lower rates were observed in Xuzhou and Suzhou in the southern region. Among the 95 districts and counties over the 11-year period, Qingjiangpu of Huaian ranked among the top 3 with the highest estimated delay rates, with values of 0.648 in 2021, 0.645 in 2014, and 0.644 in 2018. Meanwhile, from 2011 to 2013, the delay rate exhibited an increasing temporal trend, which was particularly evident in Suqian. In contrast, Figure S2 in Multimedia Appendix 1 presents the observed delay rates, which show much larger fluctuations, reflecting random noise and potential instability due to small-area sample variation. The differences between Figures S1 and S2 in Multimedia Appendix 1 arise because the Bayesian spatiotemporal Beta model smooths random noise and incorporates both spatial and temporal dependencies. By integrating relevant covariates and modeling residual correlation through random effects, it effectively adjusts for unobserved dependence and confounding, producing spatially coherent and statistically reliable estimates.

The median of the estimated TB diagnostic delay rate was 0.352. Based on the obtained median, the results from the Bayesian spatiotemporal binomial model (Table S1 in Multimedia Appendix 1) show that counties with a higher proportion of agricultural workers and higher GDP were more likely to experience high diagnostic delays. Conversely, counties with larger shares of local patients, larger resident populations, and the COVID-19 period were associated with a lower risk of high delays. Furthermore, Table S2 in Multimedia Appendix 1 indicated that GDP, TB incidence, and health care technicians had significant Granger causal effects on the temporal changes in the delay rate (*P*<.05).

### Sensitivity Analysis

The sensitivity analysis confirmed the consistency of our main results. We applied penalized complexity priors to spatial and temporal model parameters to constrain model complexity and prevent overfitting (Tables S3-S6 in Multimedia Appendix 1) [29]. Across these analyses, the direction and statistical significance of the main variables remained consistent, with a slight change in the magnitude of some coefficients. To examine the impact of risk factors associated with TB diagnostic delays over shorter periods, we divided the study period into 2 subperiods (2011‐2015 vs 2016‐2021), representing China’s 12th and 13th Five-Year Plans for Tuberculosis Prevention and Control (Table S7 in Multimedia Appendix 1) [30]. We found that the significant associations between diagnostic delay and residency or occupation observed in the first subperiod disappeared in the second subperiod, likely due to expanded health care access and continuous public health developments in Jiangsu.

## Discussion

This research analyzed 332,091 patients with TB in Jiangsu Province from 2011 to 2021, combining individual-level analysis with county-level spatiotemporal modeling to enhance understanding of diagnostic delay risk factors and their spatial and temporal patterns. At the individual level, we found that all 7 risk factors (ie, age, sex, occupation, patient source, type of diagnosis, tracking status, and type of hospital) were significant. At the county level, significant spatial clustering was observed from 2015. Drawing on such spatial dependence, we found that the proportion of local patients and the resident population were significantly and negatively associated with the TB diagnostic delay rates. Counties with higher proportions of older adults and agricultural-worker patients were more likely to experience high diagnostic delays. Moreover, GDP, TB incidence, and health care technicians exhibited significant effects on temporal changes in the delay rate.

Various individual characteristics were found to be significantly associated with the status of TB diagnostic delay. The odds of experiencing a TB diagnostic delay were significantly lower for males, consistent with the findings of previous studies [31,32]. A study in Portugal suggested that the higher overall TB burden in males (male-to-female ratio 2:1) could increase clinical suspicion and expedite diagnosis when men seek care [33]. The odds of experiencing TB diagnostic delay for education industry workers were also significantly lower, potentially attributed to strict TB screening programs for students and higher health management standards in Jiangsu and elsewhere [34]. We found older adult patients had higher odds of experiencing TB diagnostic delay, mainly due to factors such as lower education levels, poorer health awareness, lack of knowledge on TB prevention and treatment, economic difficulties, and insufficient social support [35,36]. Regarding the type of hospital, patients diagnosed by CHCs had higher odds of experiencing TB diagnostic delay, likely because these CHCs have limited resources and clinical experience [37]. For example, an artificial intelligence–assisted diagnostic platform was launched in Jiangsu Province in 2023, but this system has not been implemented at CHCs [38]. In contrast, patients diagnosed by CDCs had much lower odds of experiencing delay, underscoring the specialized knowledge needed for early TB diagnosis and thus the critical role of CDCs in TB detection. Finally, the significantly lower odds of diagnostic delay during the COVID-19 pandemic likely reflect how rigorous respiratory screening, targeting basically the same symptoms (eg, fever and cough), prompted earlier identification of TB cases that might be otherwise overlooked [2].

The spatial distribution of TB diagnostic delay exhibited clear characteristics of spatial clustering in Jiangsu Province. The global Moran *I* index showed no significant spatial autocorrelation in TB diagnostic delay rates from 2011 to 2014. Starting in 2015, however, significant spatial clustering emerged (Moran *I*=0.110-0.193; *P*<.05 for 2015-2017 and 2019-2020), indicating that the delay rates formed a stable spatial dependence pattern across counties or districts [25,39]. The mixing parameter of the Bayesian spatiotemporal Beta model was estimated at 0.318, indicating that a substantial portion of the spatial variation was attributable to structured spatial effects, also reflecting interdependency in the delay-risk patterns of neighboring areas. A possible explanation for this is the promotion of rapid drug-resistant TB molecular biological testing equipment in Jiangsu Province, which has improved TB diagnostic efficiency but may be disproportionately allocated across counties/districts due to a limited supply [40].

Our research revealed significant associations between the key factors and the TB diagnostic delay rate. For each unit increase in the proportion of local patients, the TB diagnostic delay rate decreased by 33.9%. This suggested that patients who lived permanently within the county might have had better access to local health services, greater familiarity with the health care system, or improved continuity of care, all of which could have facilitated earlier diagnosis [41]. Additionally, each increase of 100,000 residents was associated with a 2% decrease in the TB diagnostic delay rate. Larger populations were typically found in more urbanized or economically developed counties, which tended to have better health care infrastructure, higher diagnostic capacity, and more accessible TB services [42]. When counties were classified using the median estimated delay rate, those with larger older adult populations were consistently identified as high-delay areas, which was consistent with the individual-level associations. A higher proportion of agricultural-worker patients was likewise linked to greater diagnostic delays, reflecting structural barriers such as limited health care access, seasonal labor patterns, and insufficient disease awareness [43]. Panel Granger causality tests further indicated that GDP and health care technician density were key drivers of temporal fluctuations in diagnostic delay. Higher GDP likely supported more advanced diagnostic infrastructure and resource allocation, while greater technician density enhanced diagnostic capacity and accelerated case detection [44,45].

The significance of this study lies in three main aspects. First, there had been a lack of large-scale, population-based investigations into diagnostic delay in TB in Jiangsu Province. Based on a comprehensive surveillance system covering 332,091 patients with TB, the study identified several key risk factors associated with TB diagnostic delay in the general population of Jiangsu, providing an essential empirical basis for targeted interventions. Second, the analysis confirmed the existence of spatial correlation and random effects in TB diagnostic delay rates, thereby underscoring the necessity of using a Bayesian spatiotemporal modeling approach to capture the underlying spatial and temporal dependence appropriately. Third, we conducted statistical analysis at both the individual and county levels, which accounts for geographic disparities while estimating the risk factors associated with TB diagnostic delay rates. This approach leads to more targeted and synergistic public health strategies for reducing regional TB diagnostic delay rates, as well as individual chances of experiencing TB diagnostic delays.

Our study also has several limitations. First, while diagnostic delay may affect the temporal alignment between symptom onset and reporting, the calculation of TB incidence rate was not adjusted for such delays and thereby may be biased [46]. Second, due to data limitations, this study focused on the total diagnostic delay, making it impossible to distinguish between patient delay and health system delay. Future studies with more detailed health care-seeking information are needed to explore these 2 components separately. Third, continuous socioeconomic development over the decade may introduce temporal heterogeneity in the impact of risk factors, limiting the findings to this specific developmental stage.

In conclusion, a multifaceted and targeted approach is essential for effectively reducing diagnostic delays in TB. First, efforts should be made to promote proactive health-seeking behaviors among individuals, particularly among high-risk populations such as agricultural workers, older adults, and female individuals. These groups are more vulnerable to pulmonary diseases and often underrepresented in passive case detection strategies [47]. Second, from a health system perspective, it is necessary to enhance TB screening and testing protocols for migrants by improving access to care and removing systemic barriers that can delay their diagnoses, should they have TB [48]. Third, more resources should be allocated to CHCs to enhance their diagnostic capacity and knowledge of TB, as they are often the first point of contact for patients with TB, especially in underdeveloped areas [49].

## Supplementary material

10.2196/80052 Multimedia Appendix 1 Estimated and observed tuberculosis diagnostic delay rates, sensitivity analyses, Bayesian spatiotemporal binomial model results, and the panel Granger causality analysis results.

## Abbreviations

CDC: Centers for Disease Control and Prevention
CHC: community health center
GDP: gross domestic product
INLA: integrated nested Laplace approximation
OR: odds ratio
TB: tuberculosis
TBIMS: Tuberculosis Information Management System
WHO: World Health Organization
