# Supplementary material for: Risk Factors Associated With Tuberculosis Diagnostic Delay in the Jiangsu Province, China (2011-2021): Spatiotemporal Database Analysis Study
Source: JMIR Public Health Surveill. 2026 Jan 26;12:e80052. doi: 10.2196/80052 (PMC12909744; doi:10.2196/80052)
Supplement: Multimedia Appendix 1 [file publichealth-v12-e80052-s001.docx]

**Appendix**


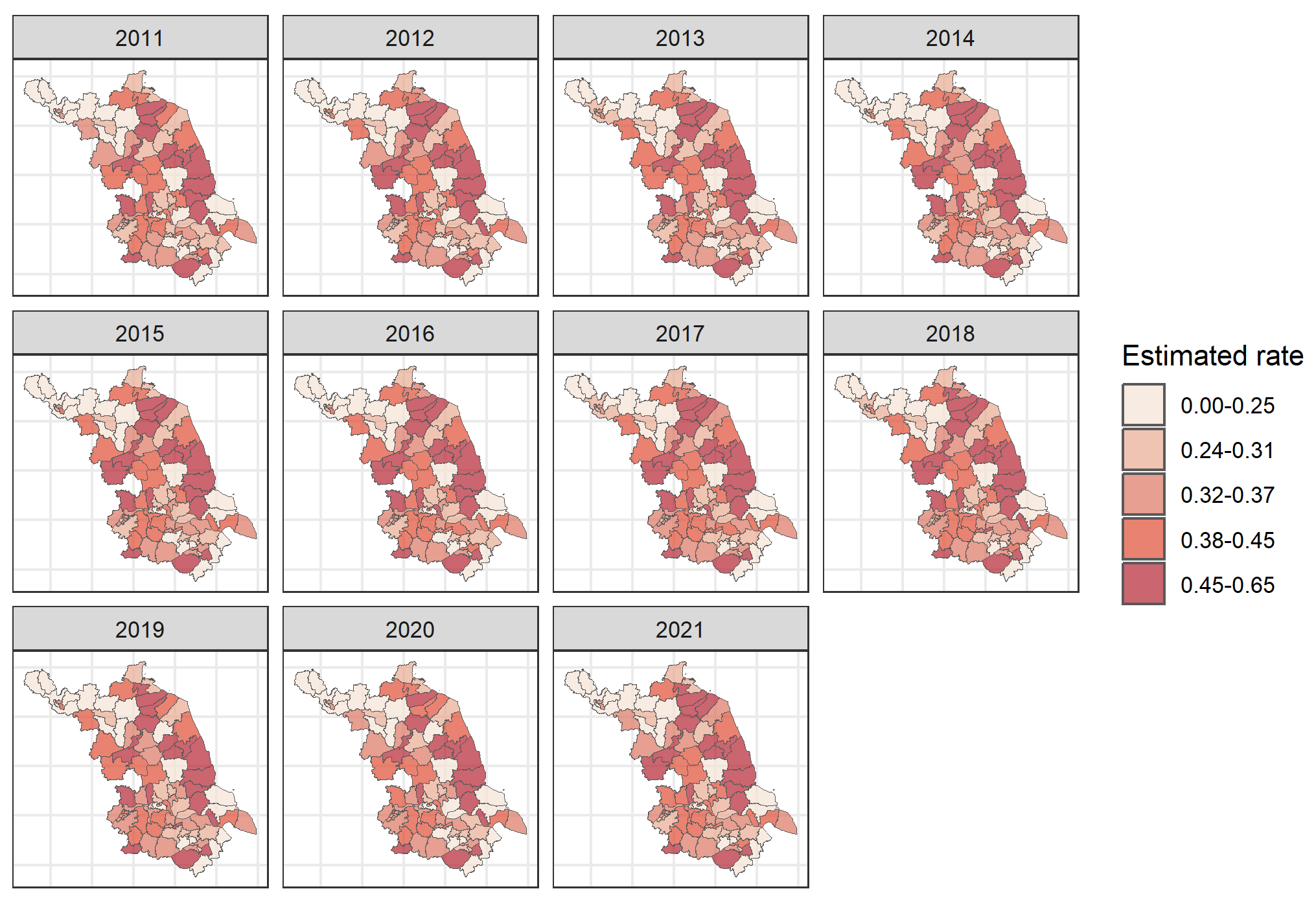


Figure S1: Spatial distribution of the estimated TB diagnostic delay rate in Jiangsu province, 2011—2021.

**
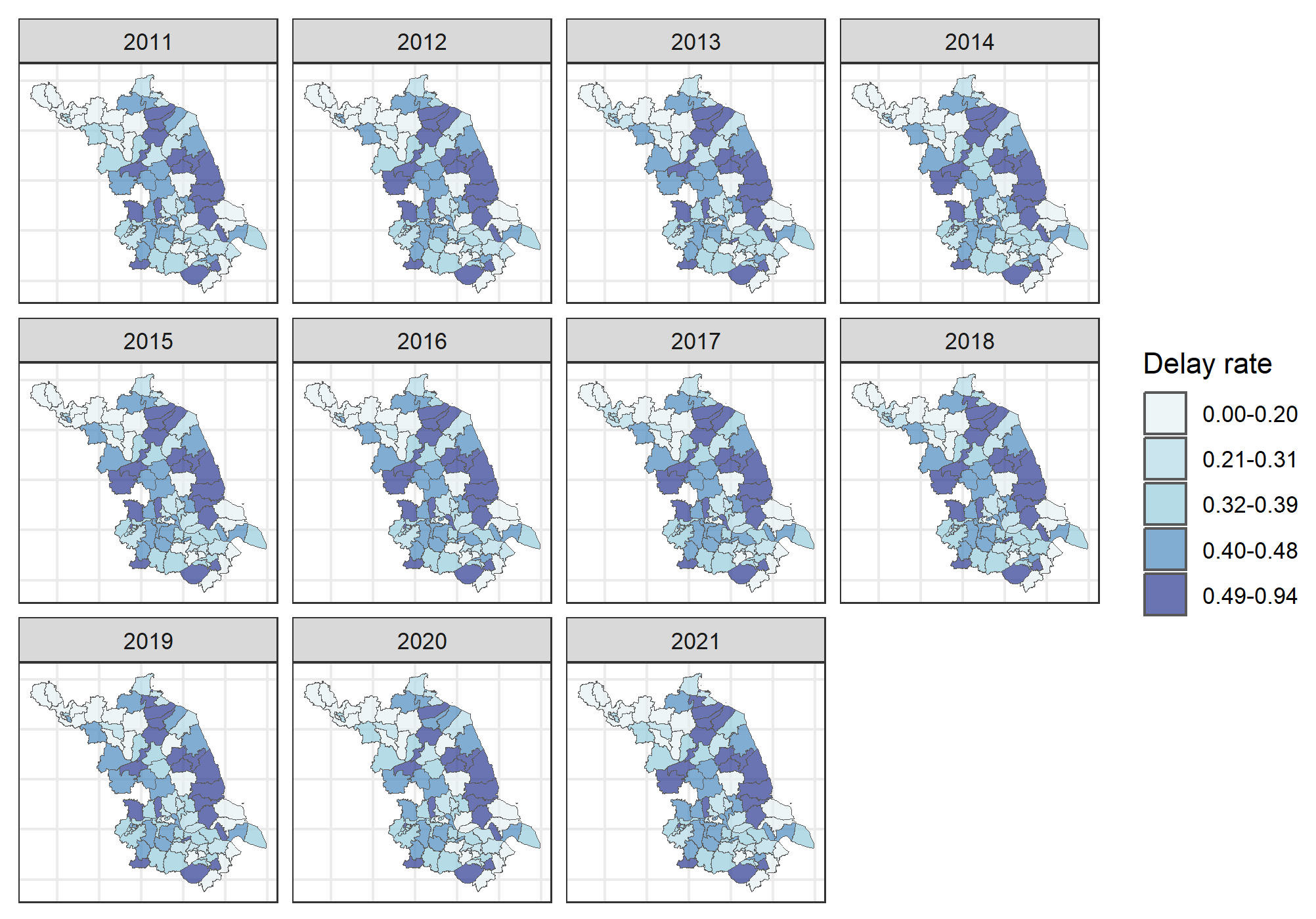
**

Figure S2: Spatial distribution of the observed TB diagnostic delay rate in Jiangsu province, 2011—2021

Table S1: The result of the Bayesian spatiotemporal binomial model identifying factors associated with high TB diagnostic delay at the county level in Jiangsu Province, 2011—2021.

| Variables | Mean | 0.025quant | 0.975quant |
| --- | --- | --- | --- |
| Proportion of elderly patients | 7.121 | $-$0.052 | 14.448 |
| Proportion of male patients | $-$0.261 | $-$0.933 | 0.409 |
| Proportion of local patients | $-$10.479 | $-$15.652 | $-$5.748 |
| Proportion of agricultural-worker patients | 12.294 | 6.121 | 18.860 |
| GDP | 2.419 | 1.344 | 3.610 |
| TB incidence rate | 0.334 | $-$1.260 | 1.919 |
| Healthcare technicians | $-$0.075 | $-$0.372 | 0.220 |
| Resident population | $-$0.517 | $-$0.809 | $-$0.243 |
| COVID-19 | $-$2.445 | $-$3.870 | $-$1.099 |

Table S2: The result of panel Granger causality analysis for factors potentially influencing the diagnostic delay rate.

| Variables | *p*-value | Granger causality (*p* < 0.05) |
| --- | --- | --- |
| Proportion of elderly patients | 0.615 | No |
| Proportion of male patients | 0.423 | No |
| Proportion of local patients | 0.453 | No |
| Proportion of agricultural-worker patients | 0.423 | No |
| GDP | 0.004 | Yes |
| TB incidence rate | 0.023 | Yes |
| Healthcare technicians | < 0.001 | Yes |
| Resident population | 0.437 | No |
| COVID-19 | 0.978 | No |

Table S3: The result of the Bayesian spatiotemporal Beta model of TB diagnostic delay rate at the county level in Jiangsu province, 2011—2021, using a prior for the BYM2 spatial effect $(P(1/\tau_{b}>1)=0.01; P(\phi<0.5)=0.5)$.

| Variables | Mean | 0.025quant | 0.975quant |
| --- | --- | --- | --- |
| Proportion of elderly patients | 0.229 | $-$0.285 | 0.742 |
| Proportion of male patients | $-$0.002 | $-$0.030 | 0.025 |
| Proportion of local patients | $-$0.415 | $-$0.691 | $-$0.139 |
| Proportion of agricultural-worker patients | 0.188 | $-$0.175 | 0.548 |
| GDP | 0.061 | $-$0.014 | 0.135 |
| TB incidence rate | 0.056 | $-$0.067 | 0.180 |
| Healthcare technicians | $-$0.009 | $-$0.024 | 0.007 |
| Resident population | $-$0.020 | $-$0.034 | $-$0.005 |
| COVID-19 | $-$0.048 | $-$0.148 | 0.053 |

Table S4: The result of the Bayesian spatiotemporal Beta model of TB diagnostic delay rate at the county level in Jiangsu province, 2011—2021, using a prior for the BYM2 spatial effect $(P(\frac{1}{\tau_{b}}>2)=0.01; P(\phi<0.8)=0.5)$.

| Variables | Mean | 0.025quant | 0.975quant |
| --- | --- | --- | --- |
| Proportion of elderly patients | 0.227 | $-$0.287 | 0.740 |
| Proportion of male patients | $-$0.002 | $-$0.030 | 0.025 |
| Proportion of local patients | $-$0.415 | $-$0.691 | $-$0.138 |
| Proportion of agricultural-worker patients | 0.184 | $-$0.180 | 0.545 |
| GDP | 0.062 | $-$0.013 | 0.136 |
| TB incidence rate | 0.056 | $-$0.067 | 0.180 |
| Healthcare technicians | $-$0.009 | $-$0.024 | 0.007 |
| Resident population | $-$0.020 | $-$0.034 | $-$0.005 |
| COVID-19 | $-$0.049 | $-$0.148 | 0.051 |

Table S5: The result of the Bayesian spatiotemporal Beta model of TB diagnostic delay rate at the county level in Jiangsu province, 2011—2021, using a prior for the RW1 temporal effect $(P(1/\tau>0.5)=0.01)$.

| Variables | Mean | 0.025quant | 0.975quant |
| --- | --- | --- | --- |
| Proportion of elderly patients | 0.170 | $-$0.361 | 0.698 |
| Proportion of male patients | $-$0.003 | $-$0.031 | 0.025 |
| Proportion of local patients | $-$0.411 | $-$0.687 | $-$0.134 |
| Proportion of agricultural-worker patients | 0.176 | $-$0.189 | 0.539 |
| GDP | 0.044 | $-$0.038 | 0.124 |
| TB incidence rate | 0.081 | $-$0.049 | 0.214 |
| Healthcare technicians | $-$0.012 | $-$0.029 | 0.005 |
| Resident population | $-$0.022 | $-$0.038 | $-$0.006 |
| COVID-19 | $-$0.041 | $-$0.183 | 0.108 |

Table S6: The result of the Bayesian spatiotemporal Beta model of TB diagnostic delay rate at the county level in Jiangsu province, 2011—2021, using a prior for the RW1 temporal effect $(P(1/\tau>2)=0.01)$.

| Variables | Mean | 0.025quant | 0.975quant |
| --- | --- | --- | --- |
| Proportion of elderly patients | 0.162 | $-$0.370 | 0.690 |
| Proportion of male patients | $-$0.003 | $-$0.031 | 0.025 |
| Proportion of local patients | $-$0.411 | $-$0.687 | $-$0.134 |
| Proportion of agricultural-worker patients | 0.173 | $-$0.193 | 0.536 |
| GDP | 0.044 | $-$0.038 | 0.124 |
| TB incidence rate | 0.083 | $-$0.048 | 0.216 |
| Healthcare technicians | $-$0.012 | $-$0.030 | 0.004 |
| Resident population | $-$0.022 | $-$0.038 | $-$0.006 |
| COVID-19 | $-$0.040 | $-$0.188 | 0.113 |

Table S7: The result of the Bayesian spatiotemporal Beta model of TB diagnostic delay rate at the county level in Jiangsu province across two distinct time periods (2011–2015 and 2016–2021).

| Variables | 2011–2015 | 2016–2021 |
| --- | --- | --- |
|  | Mean (95%CI) | |
| Proportion of elderly patients | 0.491 ($-$0.340 to 1.318) | 0.202 ($-$0.422 to 0.825) |
| Proportion of male patients | $-$0.021 ($-$0.058 to 0.016) | 0.005 ($-$0.036 to 0.046) |
| Proportion of local patients | $-$0.803 ($-$1.180 to $-$0.427) | $-$0.087 ($-$0.463 to 0.290) |
| Proportion of agricultural-worker patients | 0.656 (0.174 to 1.139) | 0.170 ($-$0.303 to 0.637) |
| GDP | $-$0.005 ($-$0.099 to 0.088) | $-$0.007 ($-$0.126 to 0.113) |
| TB incidence rate | $-$0.123 ($-$0.300 to 0.054) | 0.319 (0.057 to 0.580) |
| Healthcare technicians | 0.012 ($-$0.010 to 0.033) | $-$0.012 ($-$0.040 to 0.016) |
| Resident population | $-$0.020 ($-$0.053 to 0.013) | $-$0.007 ($-$0.026 to 0.012) |
| COVID-19 | – | $-$0.011 ($-$0.108 to 0.085) |
